# Transcranial Direct Current Stimulation (tDCS) Ameliorates Stress-Induced Sleep Disruption via Activating Infralimbic-Ventrolateral Preoptic Projections

**DOI:** 10.3390/brainsci14010105

**Published:** 2024-01-22

**Authors:** Yu-Jie Su, Pei-Lu Yi, Fang-Chia Chang

**Affiliations:** 1 Department of Veterinary Medicine, School of Veterinary Medicine, National Taiwan University, Taipei 106216, Taiwan; r10629003@ntu.edu.tw; 2 Department of Sport Management, College of Tourism, Leisure and Sports, Aletheia University, Taipei 251306, Taiwan; 3 Neurobiology and Cognitive Science Center, National Taiwan University, Taipei 106216, Taiwan; 4 Graduate Institute of Acupuncture Science, College of Chinese Medicine, China Medical University, Taichung City 404328, Taiwan; 5 Department of Medicine, College of Medicine, China Medical University, Taichung City 404328, Taiwan; 6 Graduate Institute of Brain and Mind Sciences, College of Medicine, National Taiwan University, Taipei 106216, Taiwan

**Keywords:** designer receptors exclusively activated by designer drugs (DREADDs), infralimbic cortex, sleep, stress, transcranial direct current stimulation (tDCS), ventrolateral preoptic area

## Abstract

Transcranial direct current stimulation (tDCS) is acknowledged for its non-invasive modulation of neuronal activity in psychiatric disorders. However, its application in insomnia research yields varied outcomes depending on different tDCS types and patient conditions. Our primary objective is to elucidate its efficiency and uncover the underlying mechanisms in insomnia treatment. We hypothesized that anodal prefrontal cortex stimulation activates glutamatergic projections from the infralimbic cortex (IL) to the ventrolateral preoptic area (VLPO) to promote sleep. After administering 0.06 mA of electrical currents for 8 min, our results indicate significant non-rapid eye movement (NREM) enhancement in naïve mice within the initial 3 h post-stimulation, persisting up to 16–24 h. In the insomnia group, tDCS enhanced NREM sleep bout numbers during acute stress response and improved NREM and REM sleep duration in subsequent acute insomnia. Sleep quality, assessed through NREM delta powers, remains unaffected. Interference of the IL-VLPO pathway, utilizing designer receptors exclusively activated by designer drugs (DREADDs) with the cre-DIO system, partially blocked tDCS’s sleep improvement in stress-induced insomnia. This study elucidated that the activation of the IL-VLPO pathway mediates tDCS’s effect on stress-induced insomnia. These findings support the understanding of tDCS effects on sleep disturbances, providing valuable insights for future research and clinical applications in sleep therapy.

## 1. Introduction

Insomnia, a widespread challenge in contemporary society, is defined by insufficient sleep quantity or quality, marked by difficulties in falling asleep, frequent awakenings, and post-wake fatigue [1,2]. Contributing factors such as stress, anxiety, and various mental or physical health conditions have been associated with heightened arousal in individuals contending with insomnia [3,4].

The pathophysiology of insomnia can be broadly classified into two models: the cognitive model and the physiological or neurophysiological model [3]. The cognitive model posits that life stress and worries disrupt sleep, leading to acute episodes of insomnia. Conversely, the physiological model emphasizes factors such as metabolic rate, heart rate, and neuroendocrine processes. Insomnia, seen as a dysregulation of the sleep system, involves mutual inhibition between sleep-promoting areas (e.g., ventrolateral preoptic nucleus and median preoptic nucleus) and wake-promoting areas (such as tuberomammillary nucleus, dorsal raphe, and locus coeruleus) in the sleep switch model [2,4].

The sleep switch model highlights the crucial role of the ventrolateral preoptic nucleus (VLPO) in the regulation of sleep [4,5,6,7]. Situated in the anterior hypothalamus, VLPO consists of a small group of neurons located above and to the side of the human brain’s optic chiasm. During sleep, VLPO demonstrates notably high activity, sending inhibitory signals to wake-promoting brain areas and suppressing their effects [5]. Insomnia onset has been linked to damage in the VLPO, while its activation directly promotes sleep [8,9], rendering it a focal point for numerous methods in the treatment of insomnia.

Treatment options for insomnia encompass both non-pharmacological interventions rooted in cognitive-behavioral approaches and pharmacological therapy, with widespread use of benzodiazepine receptor agonists (BzRAs) [2,10]. BzRAs, which include both benzodiazepines (BZD) and non-benzodiazepine medications, bind to gamma-aminobutyric acid (GABAA) receptors, inducing a sedative and hypnotic effect [10,11]. Multiple lines of evidence indicate that the inhibitory neurotransmitter GABA in the central nervous system (CNS) plays a key role in the etiology of insomnia [12]. It regulates brain activity, promoting relaxation by reducing neuronal excitability through the inhibition of nerve transmission [13]. These insomnia medications indeed demonstrate significant efficacy in treating insomnia, particularly by noticeably improving sleep duration. However, as insomnia tends to be a chronic condition, prolonged use of these medications may lead to the development of drug tolerance in some patients, with rebound insomnia reported upon discontinuation. Additionally, reported side effects such as dose-dependent daytime drowsiness, extended reaction times, and memory impairment further contribute to limitations in the widespread utilization of these drugs [14]. This is particularly crucial for elderly patients with compromised respiratory systems or those requiring extra caution when using benzodiazepines [15]. The quest for treatments with fewer side effects and increased effectiveness has renewed interest in an age-old therapy: transcranial direct current stimulation (tDCS) [16].

TDCS, a non-invasive brain stimulation technique, involves placing positive (anode) and negative (cathode) electrodes on the scalp and applying a weak electrical current to modulate neural activity [17]. Unlike inducing action potentials, tDCS modulates resting membrane potential, facilitating depolarization or hyperpolarization and altering neuronal excitability [18]. Research suggests that tDCS has the capacity to modulate neural plasticity, generating effects akin to long-term potentiation (LTP) and long-term depression (LTD) [19,20,21]. Anodal stimulation induces excitation, while cathodal stimulation produces inhibitory effects [18,22].

TDCS has found application in diverse psychiatric and neurological disorders, demonstrating efficacy in conditions such as major depressive disorder, drug addiction, Alzheimer’s disease, and attention deficit hyperactivity disorder (ADHD) [23]. The dorsolateral prefrontal cortex (DLPFC), crucial for executive functions, is often the targeted region in tDCS applications [24,25,26]. While tDCS has demonstrated promising potential in addressing psychiatric disorders, research suggests its potential impact on sleep. In a study involving bipolar disorder patients, after receiving daily 20-minute sessions of 2 mA electrical stimulation for three consecutive weeks, a significant reduction in the PSQI index was observed, indicating an improvement in sleep quality [27]. However, in another study focusing on patients with painful diabetic polyneuropathy (PDPN), despite pain relief, no significant differences were observed in sleep quality scores [28]. These studies collectively suggest that tDCS may indeed influence sleep; however, its effects are not consistent, and this phenomenon is also evident in its application in insomnia treatment [29].

In studies specifically targeting insomnia treatment, one study involving 19 insomnia patients indicated that tDCS administered before sleep demonstrated no changes in sleep continuity or structure [30]. Another trial with seven chronic insomnia patients, conducted as a one-month, double-blind, randomized, sham-controlled experiment, revealed improvements in total sleep time (TST) and sleep efficiency (SE) in the anodal stimulation group [31], indicating potential efficacy but underscoring the necessity for further research and exploration in the realm of insomnia treatment.

In these tDCS studies, consistent emphasis is placed on the importance of appropriately configuring the conditional parameters of an effective tDCS device. These parameters include, but are not limited to:

(a) Current Intensity and Duration: Many studies suggest that the impact of current duration and intensity on outcomes is not necessarily linear [32]. An example study indicated that excessively long stimulation times may affect the expected effects of polarity, such as anodal stimulation causing inhibition of cortical excitability or vice versa [33];
(b) Electrode Placement: For tDCS to form a complete circuit, two electrodes are required. The target electrode (anode or cathode) is placed on the specific brain region to be modulated, while the reference or counter electrode (the one with the opposite polarity to the target electrode) is placed in the corresponding area based on the hypothesis or target configuration [34,35,36]. When placing the reference electrode, it is necessary to consider the distance between the two electrodes so that the current can pass through the target brain region. If the distance between the two electrodes is too close, it may lead to a shunting effect, causing the current to dissipate through the scalp [35]. In clinical applications, especially in the field of human psychiatric disorders, selected brain stimulation areas often include the motor cortex and dorsolateral prefrontal cortex. Some research results have shown that after stimulating these regions, patients experience significant improvements in sleep quality [37] and related indicators such as sleep efficiency or number of awakenings [29,38];
(c) Current Density and Charge Density: Current density (A/m^2^) is the amount of electric charge passing through a unit area during a given period, while charge density (C/m^2^) is the multiplication of current density and the duration. In a study investigating brain tissue damage in rats following tDCS stimulation, the findings suggested that brain tissue can be harmed when both current density and charge density surpass a certain threshold [39]. This implies that even with high current density if the duration is very short, the impact on the tissue may be weak. Conversely, when applying extremely low current density and prolonging the duration, even with a high final charge density, it does not result in tissue damage. Factors influencing current density also include the electrode’s contact area with the skull, essentially the size of the electrode. Therefore, when setting the current intensity, adjustments should be made based on the electrode’s contact area used rather than using the same current intensity directly. Furthermore, reducing the impedance between the electrode and the contact surface is also of significant importance [35]. In most experiments, the primary method for ensuring conductivity involves the use of saline, while some studies employ conductive gel [34]. However, in our experiment, the extremely low current we applied led to significant resistance when using saline as the conducting solution. This resistance prevented the formation of a complete circuit. As a result, we opted for chloride-free conductive gel. This not only ensured the flow of current but also prevented any potential chemical reactions between the electrodes and the conducting solution.

Various models have been developed to simulate insomnia symptoms in animals. One approach involves inducing insomnia through drug administration, commonly using caffeine [40]. The effects and duration of this method vary based on dosage [41] and the animal strain [42]. Another prevalent model is stress-induced, with stress considered the most typical cause among various factors contributing to insomnia [43]. In humans, the “first night effect” (FNE) is a widely recognized phenomenon associated with sleeping in an unfamiliar environment for the first time. It is characterized by a reduction in total sleep duration and decreased sleep efficiency [44]. A similar phenomenon is observed in rodents. When placed in a new environment or when their cages are changed, there is an immediate delay in both non-rapid eye movement (NREM) and rapid eye movement (REM) sleep, accompanied by a decrease in the overall duration of NREM and REM sleep [42,45]. The insomnia induced by cage-exchanging has been confirmed in our research, revealing an immediate acute stress response after the bedding change, followed by acute insomnia within the subsequent hours [46].

As mentioned earlier, the impact of tDCS on sleep and its underlying mechanisms remains unclear; therefore, our study endeavors to establish a mouse tDCS model and investigate its influence on sleep and neural mechanisms. Objectives encompass the setup of the tDCS device, assessing its effects on sleep in both normal and insomnia mice, and exploring potential neural mechanisms through chemogenetic techniques. Despite the absence of a direct equivalent to the human dorsolateral prefrontal cortex (DLPFC) in mice, our hypothesis posits that tDCS can activate the infralimbic area (IL), prompting excitatory neuron conduction and subsequent activation of the ventrolateral preoptic nucleus (VLPO), thereby enhancing sleep [47,48,49]. Through this study, we aim to provide potential evidence for the mechanism of tDCS, supporting its use as a therapeutic tool for insomnia.

## 2. Materials and Methods

For our investigation, we utilized male C57BL/6 mice (20–25 g; BioLASCO Taiwan Co., Ltd., Taipei, Taiwan). The mice were housed in a temperature-controlled environment (23–24 °C) following a 12:12 light-dark cycle. Adequate food and water were available ad libitum. Post-surgery, a recovery period of at least 7 days was observed, and handling was carried out as required for experimental purposes to mitigate stress-related effects resulting from the procedures. Approval for all experimental procedures was obtained from the National Taiwan University Institutional Animal Care and Use Committee (IACUC).

### 2.1. Electroencephalography (EEG) and tDCS Electrodes Implantation

Anesthesia was induced through an intraperitoneal injection of a Zoletil (10 mg/kg, Carros, France) and xylazine (12 mg/kg, Sigma-Aldrich, St. Louis, MO, USA) mixture. Once reflexes and muscle tension loss were confirmed in the mice, the surgical area, spanning approximately from the midpoint between the eyes to the back of the ears, was shaved. Ensuring a clear airway, the mouse’s tongue was secured, and it was affixed to the stereotaxic apparatus. Following the application of eye ointment, the surgical area underwent alternating disinfection using cotton swabs soaked in povidone-iodine and alcohol (70%), with the process repeated until the cotton swab no longer retained fine hairs.

**Figure 1:**
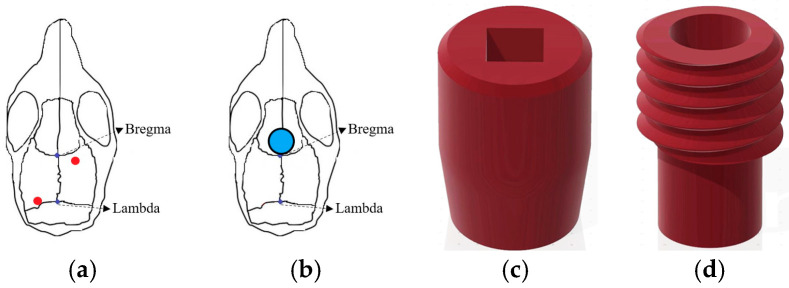
Schematic diagram of (**a**) EEG electrode position; (**b**) tDCS implant location; (**c**) tDCS electrode—cap part; (**d**) tDCS electrode—implant part. The red dot represents the EEG electrode placement, and blue circle depicts the tDCS electrode placement.

The positions of the two EEG electrodes were as follows: Anteroposterior (AP): −0.5 mm, Mediolateral (ML): +1.5 mm relative to bregma, and ML: −1.7 mm relative to lambda (Figure 1a). After drilling holes at predetermined coordinates, we affixed the EEG electrodes in their designated positions, ensuring proper contact between the electrode tips and the brain’s surface. After electrode implantation, bone cement (Tempron, GC Co., Tokyo, Japan) was meticulously applied drop by drop to cover the skull’s surface and the lower portion of the electrode. A hill-like structure was constructed layer by layer to ensure implant stability. Neomycin ointment was applied around the surgical site with a cotton swab to prevent infection. Following surgery, mice were individually housed, and ibuprofen (0.4 g/250 mL, Yung Shin Pharmaceutical Industries Co., Ltd., Taichung, Taiwan) was added to their drinking water for pain relief. Mice rested in their cages for at least one week before subsequent experimental manipulation and recording procedures.

During the surgery for implanting EEG electrodes, the tDCS electrode was concurrently placed. The stimulating electrodes were divided into two parts: the cap (Figure 1c) for installing the needle electrode and the implant placed on the mouse’s skull (Figure 1d) to connect to the cap. In the subsequent text, the term “tDCS electrode” specifically refers to the implant on the mouse’s skull. Simultaneously with EEG electrode implantation, the tDCS electrode was positioned following the aforementioned steps. However, during implant fixation, an additional step involved delicately applying super glue to its base to ensure proper positioning when covered with bone cement at the surgery’s conclusion. As illustrated in Figure 1b, the implant’s center was located at AP: +1.7 mm, relative to bregma, directly targeting the infralimbic region. The contact area between the electrode and the skull was 9.61 mm^2^, with the capacity to hold a volume of conductive solution around 80 μL. As for the reference electrode, we use the EEG electrode placed at ML: −1.7 mm relative to the lambda position to serve as the reference electrode.

### 2.2. Microinjection of Fluorogold

While the neural circuit from the infralimbic to the VLPO has been identified in rats [50], there is limited research on the relevant pathways in mice. To verify the existence of the presumed neural pathway, we injected fluorogold into the VLPO of mice. Fluorogold, a retrograde synaptic transport tracer, is widely employed for tracing neural system pathways [51,52]. After surgically exposing the mouse skull by cutting the skin, we used a drill to create an opening on the skull at coordinates AP: −0.1 mm, ML: ±0.75 mm, relative to bregma. Subsequently, we performed microinjection at coordinates AP: −0.1 mm, ML: ±0.75 mm, DV: −5.6 mm, relative to bregma, injecting 200 nL of fluorogold at a rate of 1 nL/sec and allowing an 8-min retention time. Following the injection, the opening was sealed with bone cement, and the scalp was sutured using 3/0 stitches. Subsequently, the mice were returned to individual cages to rest.

### 2.3. Designer Receptors Exclusively Activated by Designer Drugs (DREADDs) and Cre/DIO System

Designer Receptors Exclusively Activated by Designer Drugs (DREADDs) are artificially modified G-protein coupled receptors (GPCRs) primarily engineered from human muscarinic receptors. These receptors respond exclusively to specific small-molecule drugs, including clozapine-*N*-oxide (CNO), clozapine (CLZ), and Compound **21** (Cmpd-**21**) [53,54,55]. Commonly used DREADDs can be categorized into four main pathways of activation: Gq, Gi, Gs, and β-arrestin. Our research specifically focuses on inhibitory Gi-DREADDs, particularly hm4Di, derived from the muscarinic M4 receptor. Activation by CNO or the mentioned drugs initiates inhibitory G protein (Gi) signaling cascades, thereby suppressing neural signal transmission and excitability [56,57].

To achieve cell-type-specific regulation, we combined DREADDs with the Cre/DIO system. Cre (Cyclization Recombination Enzyme) is a recombinase originally discovered in the P1 bacteriophage, recognizing LoxP sites in DNA sequences and facilitating genetic recombination between two LoxP sites [58]. DIO (Double-Floxed Inverted Open reading frame), also known as a gene switch FLEX based on LoxP sites, activates gene expression through Cre-mediated recombination. To explore the excitatory neural pathway from the IL to VLPO, we injected pENN.AAV.CamKII.HI.GFP-Cre.WPRE.SV40 into the IL. This specific viral vector, a gift from James M. Wilson, was selected for its CamKII promoter properties, anticipating the infection and expression of GFP-Cre in excitatory neurons projecting from the IL. Paired with it is pAAV-hSyn-DIO-hM4D(Gi)-mCherry (AAVrg), a gift from Bryan Roth. This virus, injected into the VLPO, carries a Cre-dependent hM4D(Gi) receptor, allowing infection only in cells expressing Cre. Upon activation through clozapine (CLZ), it facilitates the blockade of the neural pathway projecting from the IL to VLPO.

The surgical procedure for virus injection, akin to EEG implantation, involved creating openings in the mouse’s skull. In addition to the two EEG electrode locations, four holes were made on both sides of the VLPO and both sides of the IL region specifically for virus injection. A glass capillary was employed for microinjection due to the relatively small size of the targeted brain regions. AAV-hSyn-DIO-hM4D(Gi)-mCherry (AAVrg) was injected into the ventrolateral preoptic area and pENN.AAV.CamKII.HI.GFP-Cre.WPRE.SV40 was injected into the IL region. Injection coordinates and volumes were precisely controlled, and after completion, injection holes were filled with bone cement. Subsequently, the installation surgery for tDCS electrodes and EEG electrodes was performed, and mice were individually housed. To allow the virus to fully infect the IL-to-VLPO pathway, mice were given an eight-week rest period before commencing subsequent experimental procedures and EEG recordings.

### 2.4. EEG Recording and Analysis

The day before commencing EEG recording, we affixed the EEG electrodes to the mouse’s head and connected them to recording wires, allowing for acclimation to the wired condition. Subsequently, the collected EEG signals underwent a 10,000-fold amplification using a Colbourn Instruments amplifier (Lehigh Valley, PA, USA; model V75-01) and were then filtered to preserve analog signals within the 0.1 to 40 Hz range. Following this, the filtered signals were converted into digital signals at a rate of 128 Hz using a signal converter (NI PCI-6033E, National Instrument, Austin, TX, USA). The obtained signals were then displayed on a computer and manually analyzed using ICELUS^®^ software (Dr. M. OPP, University of Colorado Boulder, Boulder, CO, USA).

Mouse sleep stages, categorized into wakefulness, non-rapid eye movement (NREM), and rapid eye movement (REM) sleep, were delineated during the EEG analysis [59]. Each state exhibited distinct features in peak frequency and corresponding amplitudes. Our analysis was performed in 12 s intervals, referred to as epochs, utilizing Fourier frequency transformation to extract frequency-domain features within each epoch. This methodology facilitated the capture of primary frequencies during wakefulness, NREM sleep, and REM sleep.

During wakefulness, mice exhibited a combination of delta (0.5–4.0 Hz) and theta (6.0–9.0 Hz) waves in the EEG, indicating diverse activation across brain regions. The NREM stage displayed synchronized brain activity predominantly characterized by delta waves, with this stage showing the highest amplitude among the three stages. REM sleep featured brain activity resembling wakefulness but with predominant theta waves, distinguishing it from the mixed frequencies observed during wakefulness. Using these criteria, we conducted an analysis of the percentage of each sleep stage and sleep architecture within the recording period. Furthermore, we assessed the sleep quality of mice based on delta power [60].

### 2.5. Brain Slice Preparation

After concluding experimental procedures, mice were administered intraperitoneal anesthesia to induce a deep anesthetic state. Upon confirming the absence of all reflexes, a thoracotomy was performed to initiate perfusion. Initially, blood in the mouse’s body was replaced with a phosphate-buffered saline (PBS) solution. Using a butterfly needle inserted into the left ventricle, the right atrium was incised, and perfusion continued until the liquid flowing from the right atrium became transparent. Subsequently, the PBS was replaced with 4% formalin (P6148, Sigma-Aldrich, St. Louis, MO, USA), perfusing until the entire mouse body became rigid, completing the perfusion step.

After perfusion, the mouse’s brain was extracted and submerged in 4% formalin at room temperature for one day. Subsequently, the brain was moved to a 20% sucrose solution dissolved in 0.1% phosphate buffer (PB) and kept at 4 °C for one day before being transferred to a 30% sucrose solution for an additional day. This sequential process ensures thorough dehydration of the brain, preventing the formation of ice crystals and tissue damage during subsequent frozen sectioning.

After completing the dehydration process, the brain was embedded in an optimal cutting temperature (OCT) compound (Sakura Finetek USA, Inc., Torrance, CA, USA) and preserved at −80 °C for subsequent frozen sectioning. The prepared brain tissue will be sliced into sections with a thickness of 40 μm. These sections will then be mounted on glass slides and examined under a fluorescent microscope. Since the viruses and neural tracers we selected are inherently fluorescent, no additional staining procedures will be conducted.

### 2.6. Experimental Protocols

#### 2.6.1. Establishing a Suitable tDCS Parameters

To enhance the mouse tDCS model parameters for impacting sleep, our primary goal in the initial experiment is to ascertain a current intensity that is both safe and effective. According to Liebetanz et al., a rat lesion threshold is reported at 52,400 C/m^2^ [31]. In reverse calculation, taking into account the contact area of our tDCS electrode with the skull (9.61 mm^2^), we determined a maximum safe current intensity of 839.24 μA. Notably, this aligns with the customary values employed in conventional mouse studies [36,61].

In the preliminary assessments using a 0.2 mA current, mice displayed apparent signs of itching and discomfort. This discomfort was likely linked to the placement of the EEG electrodes, acting as the reference (cathode) electrode, situated farther from the tDCS electrode. As a response, we methodically decreased the current intensity. Eventually, at 0.06 mA or lower, mice exhibited no disruption in ongoing activities, such as foraging or sleeping, although occasional itching persisted. Consequently, we designated 0.06 mA as the configuration for testing the tDCS effect. In the process of applying current, in addition to the values set by the machine, we also used an additional ammeter to confirm that the output current was constant. Additionally, if a circuit were not formed during the application of current, the machine would not continue to generate current. This is why we used conductive gel to reduce resistance. Through these methods, we could ensure that the current entered the brain with a fixed intensity, forming a circuit.

In anticipation of evaluating the potential effects of this current intensity on mice, one day prior to the commencement of baseline recording and stimulation, we pre-loaded the conductive gel into the tDCS implant on the mouse’s head and affixed the cover with connected wires. This procedure enabled the mice to acclimate to the weight of the cover and the potential discomfort resulting from contact with the conductive gel. The tDCS settings were consistently set at a current intensity of 0.06 mA, administered for a duration of 8 min.

**Figure 2:**
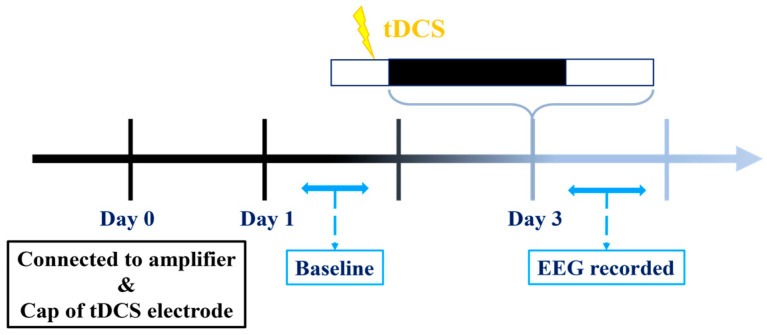
Experimental protocol for testing the effect of tDCS.

Our experimental procedures are illustrated in the diagram provided (Figure 2). EEG recordings on the first and third days commence during the dark cycle and extend for a duration of 24 h, while tDCS stimulation concludes prior to the onset of the dark cycle on the third day. Initiating recording at the beginning of the dark cycle is intentional, as our hypothesis posits that mice typically experience reduced sleep during this period compared to the light cycle. This strategic choice improves our capacity to identify potential variations in sleep patterns if tDCS exerts any influence.

#### 2.6.2. tDCS Effects on Stress-Induced Insomnia Model

In our investigation, we chose the stress-induced insomnia model for its operational simplicity and its capacity to mimic conditions akin to acute insomnia in humans. Expanding upon the insights gained from the initial experiment, where we examined the influence of anodal stimulation at a current intensity of 0.06 mA for 8 min on sleep, our objective was to explore whether these parameters could potentially have a therapeutic impact on insomnia in mice.

**Figure 3:**
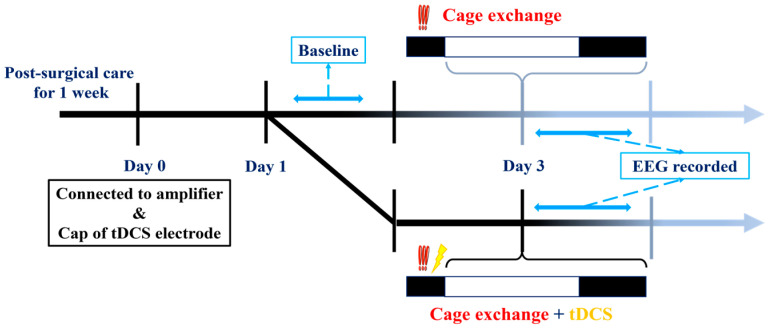
Experimental protocols for determining tDCS effects on stress-induced insomnia mice.

For this experiment, EEG recordings commenced at the beginning of the light cycle. After baseline EEG values were recorded, mice were divided into two groups. The first group constituted the insomnia group, where a mouse was transferred to a cage already occupied by another mouse a week prior to the initiation of EEG recording. The introduction of the other mouse’s odor and the unavoidable nature of the new environment triggered an immediate acute stress response phase in the relocated mouse, resulting in acute insomnia over the subsequent hours. In contrast, the second group, the treatment group, underwent tDCS therapy in the modified environment following the cage change; the stimulation was performed before the beginning of the light cycle. The comprehensive process is illustrated in the diagram below (Figure 3).

#### 2.6.3. Validate the Projection from IL to VLPO

As mentioned earlier, the pathway from the IL to the VLPO has not been thoroughly investigated. Thus, the aim of this experiment is to validate the existence of this pathway in the mouse brain. The methodology included injecting the retrograde tracer Fluorogold into the VLPO. After an 8-week post-surgery period, mice were euthanized, and their brains were meticulously dissected, sectioned, and mounted on slides. Subsequently, using a fluorescent microscope, we scrutinized whether the Fluorogold tracer retrogradely travels from the VLPO to the IL brain region. Three mice were used in this experiment.

#### 2.6.4. Determine the Effect of tDCS on Stress-Induced Insomnia Is Mediated by the IL-VLPO Pathway

To delve deeper into the potential contribution of the IL-to-VLPO pathway to tDCS-induced improvements in sleep, we utilized chemogenetic modulation of this circuit. After surgery, an 8-week period was allotted to ensure comprehensive viral infection along this pathway. The activation of hM4D(Gi) was initiated by injecting Clozapine, leading to the inhibition of excitatory neurons from the IL to VLPO. If the mechanism by which tDCS enhances sleep aligns with our hypothesis, the impact of tDCS should be hindered when this pathway is suppressed.

Between the surgery and the actual recording sessions, we implemented daily handling sessions to habituate the mice to the forthcoming Clozapine injection procedure. The day preceding the formal recording, we connected the EEG to the amplifier, filled the tDCS implant with conductive gel, and affixed the cap to acquaint them with the upcoming procedures and recording. Baseline sleep states were then recorded on the first day.

On the third day, mice underwent three distinct procedures in a randomized order:

1. The first group received an intraperitoneal injection of saline. After a 15 min interval, the mouse was relocated to a cage already occupied by another mouse to induce insomnia. EEG recording commenced after tDCS stimulation;
2. The second group received an intraperitoneal injection of clozapine (clz, 0.4 mg/kg) to inhibit the activation of the IL to VLPO pathway [55]. Fifteen minutes after the intraperitoneal injection, we conducted cage exchanging and initiated tDCS stimulation with parameters consistent with the previous settings. EEG recording began after completing the stimulation;
3. The third group received an intraperitoneal injection of saline. After a 15 min interval, they underwent cage exchanging, followed by EEG recording.

**Figure 4:**
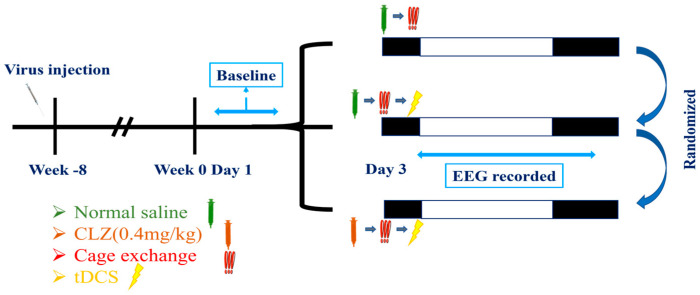
Experimental protocols for modulating the IL-VLPO pathway to assess the impact of tDCS on stress-induced insomnia.

In this experiment, each mouse experienced a minimum one-week interval between different procedures to safeguard against potential influences from preceding cage-exchanging operations and tDCS effects on subsequent experiments. The initiation of all EEG recordings occurred during the light cycle, and all specified procedures were concluded before transitioning from the dark cycle to the light cycle. The detailed procedure is depicted in the Figure below (Figure 4).

### 2.7. Statistical Analysis

The statistical analysis was carried out using SPSS version 25.0 (IBM, SPSS, Armonk, NY, USA). In the first experiment, paired *t*-tests were employed to assess differences in sleep proportions and sleep structure before and after administering tDCS to mice. For the second and third experiments, mixed-effects ANOVA was employed with different treatment groups considered as fixed effects and time points as random effects. Mauchly’s sphericity test was applied to assess the conformity of the data to the sphericity assumption. In case of non-compliance, the Greenhouse–Geisser correction was utilized. Additionally, Levene’s test for homogeneity of variances was conducted for each time point. Sleep proportion differences between groups at specific time points, sleep percentages during particular periods, and sleep structure were subjected to statistical analysis using one-way ANOVA with Bonferroni post hoc tests.

## 3. Results

### 3.1. Optimal Configuration of tDCS for Mice

#### 3.1.1. Parameters of tDCS

In the process of refining parameters to alleviate intense itching and discomfort for the mice, we commenced testing at a current intensity of 0.2 mA, gradually diminishing it. Ultimately, we found that at a current stimulation of 0.06 mA, mice experienced minimal disturbance to their ongoing activities, such as drinking water, eating, or exploring their environment. Encouraged by these favorable observations, we proceeded to examine whether continuous stimulation at this intensity for 8 min would result in tissue damage.

**Figure 5:**
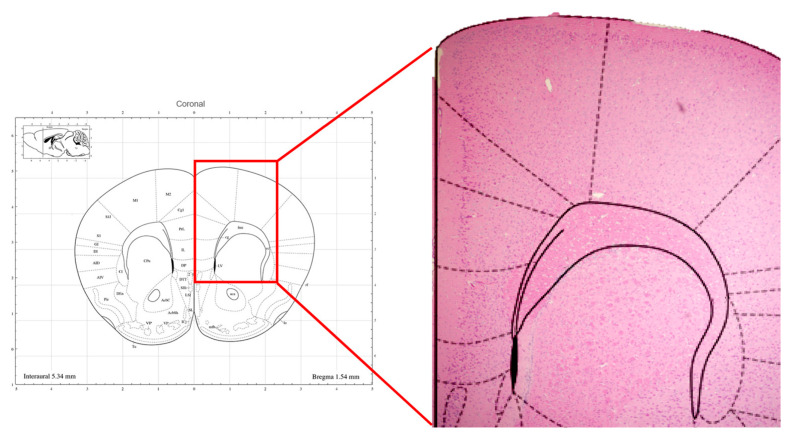
Brain tissue sections positioned directly beneath the stimulation electrode.

Histological examination revealed significant findings, as illustrated in Figure 5. Specifically, we observed that the tissue directly beneath the anode of tDCS remained intact, providing evidence of the safety of our chosen parameters. Importantly, no conspicuous signs of discomfort were noted in the mice throughout the stimulation process, affirming the overall well-tolerance of the selected current intensity and duration.

#### 3.1.2. The Influence of tDCS on Sleep in Naïve Mice

**Figure 6:**
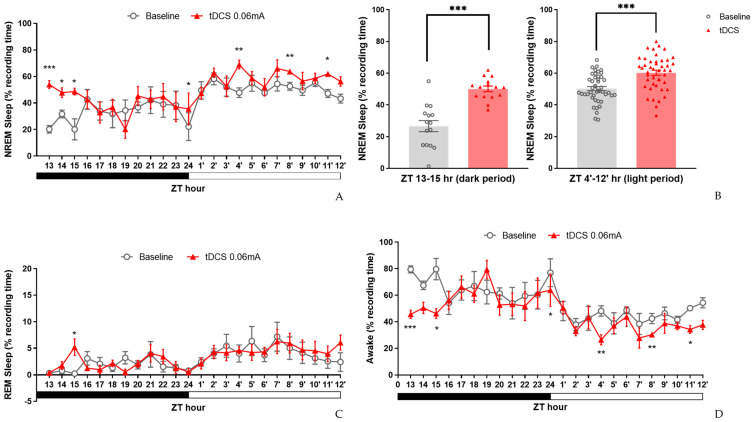
Effects of tDCS on sleep in naïve mice. (**A**) tDCS demonstrated NREM sleep enhancements at the beginning of dark period ZT13, 14, 15 and the following light phase at ZT24, 4′, 8′, 11′; (**B**) when comparing the two specific periods, ZT13−15 and ZT4′−12′, tDCS indicated significant increases of NREM sleep in both time periods; (**C**) a significant increase in REM sleep was observed at ZT15 comparing to baseline; (**D**) effect of tDCS on wakefulness in naive mice. tDCS reduced wakefulness at ZT13, 15, 24, 4′, 8′, and ZT11′. (* *p* < 0.05, ** *p* < 0.01, *** *p* < 0.001, paired *t*-test). Data are presented as the mean ± SEM. *n* = 5; ZT: zeitgeber time.

To evaluate the impact of tDCS stimulation on sleep in normal mice under the specified conditions, we conducted statistical analyses for each hour. The results revealed a significant enhancement of non-rapid eye movement (NREM) sleep in the tDCS-stimulated group (Figure 6A). Notably, significant NREM sleep enhancements were observed at ZT13 after stimulation cessation (t_(4)_ = −9.467, *p* < 0.001), ZT14 (t_(4)_ = −2.923, *p* = 0.043), and ZT15 (t_(4)_ = −3.376, *p* = 0.028). These effects persisted into the subsequent light phase at ZT24 (t_(4)_ = −3.176, *p* = 0.034), ZT4′ (t_(4)_ = −6.982, *p* = 0.002), ZT8′ (t_(4)_ = −4.717, *p* = 0.009), and ZT11′ hours (t_(4)_ = −3.868, *p* = 0.018) compared to the baseline.

Considering the time points influenced by tDCS, its effects can be broadly categorized into two periods: the initial three hours after the end of stimulation (ZT13–15) and the subsequent period during which NREM sleep continues to increase (ZT4′–12′) (Figure 6B). Significant differences between the two groups were observed during these time spans. Following tDCS administration, there was an immediate increase in the percentage of NREM sleep (t_(15)_ = −5.996, *p* < 0.001). This effect persisted during subsequent periods ZT4′–12′ (t_(44)_ = −4.82, *p* < 0.001), indicating that the impact of tDCS on promoting NREM sleep in normal mice extends beyond the initial 3 h and persists for up to 16 h after the end of stimulation.

In the analysis of REM sleep, a significant difference was observed only at the third hour (ZT15) after tDCS stimulation compared to the baseline (t_(4)_ = −2.905, *p* = 0.044), while the remaining time points did not show any significant differences (Figure 6C). Regarding wakefulness percentages, comparisons between the tDCS group and the baseline group revealed a significant decrease in wakefulness immediately following stimulation. Specifically, at ZT13 (t_(4)_ = 9.508, *p* = 0.001) and ZT15 (t_(4)_ = 3.666, *p* = 0.021) post-stimulation cessation, there was a notable reduction in wakefulness. Similarly, in the subsequent hours at ZT24 (t_(4)_ = 2.871, *p* = 0.045), ZT4′ (t_(4)_ = 4.74, *p* = 0.009), ZT8′ (t_(4)_ = 4.781, *p* = 0.009), and ZT11′ (t_(4)_ = 4.208, *p* = 0.014), the decrease in wakefulness percentage persisted and remained evident (Figure 6D).

#### 3.1.3. Effects of tDCS on Sleep Architecture and Quality

To investigate the reasons behind the increased NREM sleep and evaluate any changes in sleep quality further, we conducted an analysis of sleep architecture and NREM delta power. The results, as depicted in Table 1, revealed a notable rise in the duration of NREM bouts within the initial 12 h following tDCS application (t_(4)_ = −2.779, *p* = 0.05). Upon a more detailed examination of the two specific periods, we noted a significant increase in both the duration of NREM bouts (t_(4)_ = −3.110, *p* = 0.036) and the number of REM bouts (t_(4)_ = −3.162, *p* = 0.034) during ZT13–15 h. Similarly, in the subsequent ZT4′–12′ hours, there was a notable increase in the duration of NREM bouts (t_(4)_ = −3.129, *p* = 0.035).

**Figure 7:**
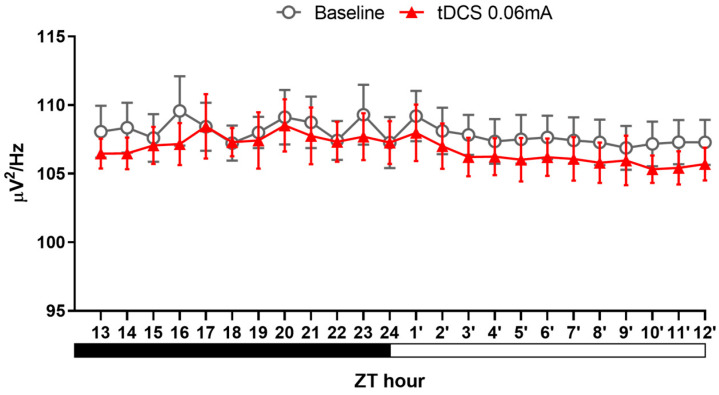
NREM delta power after tDCS treatment in naïve mice.

Concerning sleep quality (Figure 7), we did not observe significant changes in NREM delta power between the two groups at any time points. These findings not only illustrate that the parameters we established can enhance sleep but also indicate that their impact exceeds our initial expectations. Remarkably, only 8 min of stimulation could influence sleep for nearly 24 h. Furthermore, based on the analysis of sleep architecture it suggests that the heightened NREM sleep is primarily attributed to the prolonged duration of each bout. Additionally, in the initial three hours following stimulation cessation, there is an observed increase in the number of REM bouts.

### 3.2. The Therapeutic Effect of tDCS in Stress-Induced Insomnia Mice

#### 3.2.1. Sleep Modifications in Stress-Induced Insomnia Mice following tDCS Intervention

In the initial experiment, tDCS showcased its sleep-promoting effects in naïve mice. To evaluate its therapeutic potential in stress-induced insomnia mice, we applied the same tDCS parameters. In this experiment, due to recording issues, the baseline data for one mouse is missing; therefore, the *n*-value for the baseline group is 11. Our results revealed a significant interaction between group and time during the ZT1–12 period (F_(10.534, 105.336)_ = 4.903, *p* < 0.001). However, the interaction effect during the ZT13–24 period was not statistically significant.

Post-hoc analysis within the initial 12 h revealed a significant decrease in NREM sleep percentage in the bedding exchange (Bex) group compared to the baseline group (*p* < 0.001), while tDCS prevented the stress-induced reduction in NREM sleep (*p* = 0.012). These findings suggest that the NREM sleep changes induced by bedding exchange primarily occurred within the first 12 h, and tDCS treatment significantly improved NREM sleep in mice of the Bex group.

**Figure 8:**
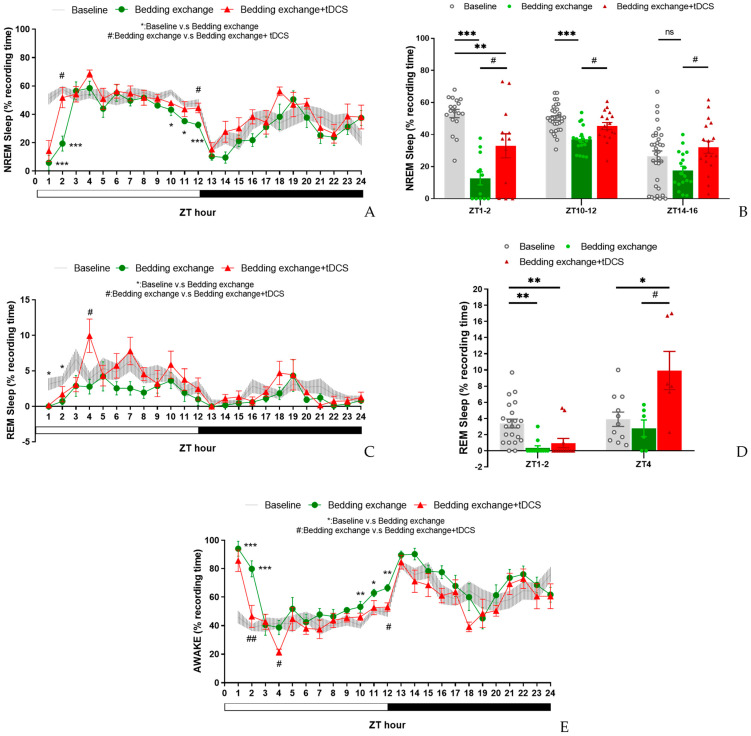
Effects of tDCS on sleep in stress-induced insomnia mice. (**A**) The decrease in NREM sleep in mice caused by bedding exchange primarily occurs in the first 12 h, distinguishing between the acute stress response phase (ZT1–2) and the subsequent acute insomnia phase (ZT10–12). When compared to the Bex group, the group treated with tDCS significantly enhanced NREM sleep in both time periods; (**B**) during the ZT1–2, ZT10–12, and ZT14–16 periods, the Bex group consistently exhibited significantly lower NREM sleep compared to the baseline group. Moreover, the Bex + tDCS group demonstrated a reversal of insomnia effects across all time intervals; (**C**) after bedding exchange, mice exhibited a significant decrease in REM sleep during the first two hours, with a trend of overall lower percentages compared to baseline in subsequent time points. In the tDCS-treated group, no statistical difference from the Bex group was observed during the first two hours, but a significant increase occurred at ZT4; (**D**) during ZT1–2, the Bex group showed a lower percentage of REM sleep, and there was no reversal effect after the tDCS administration. At ZT4, the tDCS group exhibited a higher REM sleep percentage compared to both the Bex and baseline groups; (**E**) we noticed a notable rise in wakefulness percentage for the Bex group during the ZT1–2 and ZT10–12 periods compared to the baseline, indicative of acute stress response and acute insomnia phases. Conversely, in the Bex + tDCS group, a significant reduction in wakefulness percentage was evident at ZT2, lasting until the fourth hour. Additionally, at ZT12, the Bex + tDCS group demonstrated a significant decrease in wakefulness percentage compared to the Bex group. (* *p* < 0.05, ** *p* < 0.01, *** *p* < 0.001 vs. Baseline, # *p* < 0.05, ## *p* < 0.01 vs. Bex; Repeated-measures ANOVA followed by the Bonferroni test). Data are presented as the mean ± SEM. The sample sizes for the baseline, Bex, and Bex + tDCS groups are 11, 6, and 6, respectively; ZT: zeitgeber time.

Further analysis revealed a significant decrease in non-rapid eye movement (NREM) sleep in the Bex group during ZT1 (*p* < 0.001), ZT2 (*p* < 0.001), ZT10 (*p* = 0.01), ZT11 (*p* = 0.034), and ZT12 (*p* = 0.003) (Figure 8A). Therefore, we could divide these periods into two distinct stages: ZT1–2 (representing the acute stress response phase) and ZT10–12 (representing the acute insomnia phase). To assess the effectiveness of tDCS during the bedding exchange-affected period, we focused on the two periods mentioned. Statistical analyses indicated significant differences between groups during both the ZT1–2 period (F_(2, 43)_ = 24.329, *p* < 0.001) and ZT10–12 (F_(2, 66)_ = 14.519, *p* < 0.001).

Post hoc comparisons during ZT1–2 (Figure 8B) showed a significant decrease in NREM sleep in the Bex group compared to the baseline group (*p* < 0.001). Following tDCS administration, the Bex + tDCS group exhibited a significant improvement in NREM sleep (*p* = 0.015), demonstrating the efficacy of tDCS in ameliorating NREM sleep decrease during the acute stress response phase. In ZT10–12, a significant decrease in NREM sleep in the Bex group compared to the baseline group was observed (*p* < 0.001). The tDCS treatment showed a significant NREM sleep improvement compared to the Bex group (*p* = 0.011). Notably, during this period, the NREM sleep in the tDCS treatment group showed no significant difference compared to the baseline group, suggesting the enduring therapeutic effects of tDCS are sufficient to reverse acute insomnia induced by cage exchange. During the dark cycle, although the analysis of the entire ZT13–24 period did not reveal statistical differences between groups, further analysis at ZT14–16 showed significant differences (F_(2, 66)_ = 3.659, *p* = 0.031). The decrease in NREM sleep in the Bex group did not differ significantly from the baseline, but the tDCS group exhibited significantly higher NREM sleep compared to the Bex group (*p* = 0.018). This result confirms the enduring effect of tDCS in promoting NREM sleep in mice subjected to bedding exchange, lasting at least until the 16th hour post-stimulation.

In the REM sleep analysis (Figure 8C), a significant interaction effect between groups and time was observed during ZT1–12 (F_(11.822, 118.22)_ = 28.284, *p* = 0.035). However, no significant interaction effect was found during ZT13–24. No statistically significant inter-group differences were observed in both time periods. Yet, given indications from prior experiments regarding tDCS effects on REM sleep in naïve mice at specific time points, we examined the simple main effect at those time points. Results showed significant group differences at ZT1 (F_(2, 20)_ = 6.354, *p* = 0.007), ZT2 (F_(2, 20)_ = 3.968, *p* = 0.035), and ZT4 (F_(2, 20)_ = 6.540, *p* = 0.007). Further analyses (Figure 8D) revealed that at ZT1–2, the Bex group exhibited a significant decrease in REM sleep compared to the baseline group (*p* = 0.001), and the tDCS group also showed a significant decrease compared to baseline (*p* = 0.006). However, no statistically significant differences were observed between the Bex group and the tDCS-treated group at this time period. By ZT4, REM sleep in the Bex group was no longer statistically different from the baseline, whereas the tDCS group exhibited a significant increase in REM sleep compared to the baseline (*p* = 0.016) and the Bex group (*p* = 0.012). These results highlight a phenomenon similar to the first experiment, wherein REM sleep consistently increased in the second hour following stimulation, reaching a peak at the fourth hour before gradually returning to baseline. This effect was substantial enough to counteract the impact of stress imposed by the bedding exchange.

In the wakefulness percentage analysis (Figure 8E), a significant interaction between group and time was observed during the ZT1–12 period (F_(10.439, 104.388)_ = 4.342, *p* < 0.001), with no significant interaction during the ZT13–24 period. Significant inter-group differences were detected during ZT1–12 (F(_2, 20)_ = 19.228, *p* < 0.001), with no significant differences observed during ZT13–24. Post hoc comparisons indicated that, within the ZT1–12 period, the wakefulness time in the Bex group was significantly higher than the baseline group (*p* < 0.001) and the tDCS-treated group (*p* = 0.002). However, no significant differences were observed between the Bex with tDCS group and the baseline group. This finding aligns with the trend observed in the NREM analysis, suggesting that the increase in wakefulness induced by bedding exchange can be reduced to a level not significantly different from the baseline after receiving tDCS. In the exploration of simple main effects analysis for the time factor, significant group differences emerged at various time points, including ZT1 (F_(2, 20)_ = 23.99, *p* < 0.001), ZT2 (F_(2, 20)_ = 20.023, *p* < 0.001), ZT4 (F_(2, 20)_ = 8.114, *p* = 0.003), ZT10 (F_(2, 20)_ = 5.838, *p* = 0.01), ZT11 (F_(2, 20)_ = 4.223, *p* = 0.03), and ZT12 (F_(2, 20)_ = 8.473, *p* = 0.002). Comparisons between the Bex and baseline groups revealed a significant increase in wakefulness percentage at ZT1 (*p* < 0.001), ZT2 (*p* < 0.001), ZT10 (*p* = 0.009), ZT11 (*p* = 0.031), and ZT12 (*p* = 0.002), consistent with the affected periods in NREM sleep. Conversely, when comparing the Bex with the tDCS group to the Bex group, a significant decrease in wakefulness percentage was observed at ZT2 (*p* = 0.001), ZT4 (*p* = 0.024), and ZT12 (*p* = 0.035). Notably, the group differences observed at ZT4 can be attributed to a significant increase in REM sleep at this time point, leading to a significant decrease in wakefulness percentage in the tDCS-treated group.

#### 3.2.2. Alterations of Sleep Architectures between Groups during Different Phases

In the examination of sleep architecture (Table 2), significant differences were found in wake bout duration during ZT1–12 (F_(2, 20)_ = 9.524, *p* = 0.001). Post hoc analysis revealed increased wake bout duration in the Bex group compared to the baseline group (8.7 ± 1.3 vs. 3.1 ± 0.4, *p* = 0.001), while the Bex + tDCS group (5.6 ± 1.4) showed no statistical difference from both. During ZT13–24, the Bex group exhibited a significantly lower REM bout number compared to the baseline group (0.6 ± 0.1 vs. 1.0 ± 0.1, *p* = 0.04), while the Bex + tDCS group (0.9 ± 0.1) showed higher but non-significant differences.

In the acute stress response phase (ZT1–2), the observed bout numbers of the baseline group for NREM, wake, and REM were 12.9 ± 1.9, 7.9 ± 0.9, and 2.0 ± 0.2, respectively. In contrast, the Bex group displayed significantly lower bout numbers for NREM (2.2 ± 0.7), wake (1.8 ± 0.5), and REM (0.3 ± 0.2) compared to baseline, with statistical differences of *p* = 0.001, *p* = 0.003, and *p* < 0.001, respectively. Indicating a notable impact on sleep architecture following the bedding exchange. The Bex + tDCS group exhibited slightly higher bout numbers for NREM (7.5 ± 1.8), wake (5.6 ± 1.6), and REM (0.4 ± 0.3) compared to the Bex group. While there were no statistical differences in NREM and wake compared to the other two groups, REM, despite a slight increase compared to the Bex group, remained significantly lower than the baseline (*p* < 0.001). This suggests that the Bex + tDCS group, while showing an increase, still maintained a lower level compared to the baseline group. Regarding wake bout duration, the Bex group exhibited a significantly higher duration compared to the baseline group (34.5 ± 7.0 vs. 3.3 ± 0.5, *p* < 0.001), while the Bex + tDCS group showed a reduction in the average duration of each wake bout (19.2 ± 7.0) after tDCS, with no statistical difference from the baseline group. Concerning REM bout duration, the Bex group displayed a significant decrease compared to the baseline group (0.1 ± 0.1 vs. 1.0 ± 0.1, *p* = 0.001) and the Bex + tDCS group (0.3 ± 0.2), although showing a slight increase compared to the Bex group, did not exhibit statistical differences and remained significantly lower than the baseline (*p* = 0.008).

During ZT4, the Bex + tDCS group showed increased REM bout numbers and duration compared to the Bex group (4.5 ± 0.9 vs. 1.8 ± 0.7 and 1.4 ± 0.2 vs. 0.6 ± 0.2) with statistical differences of *p* = 0.026 and *p* = 0.048, respectively. In terms of wake bout duration, the tDCS group exhibited decreased wake bout duration compared to Bex (1.1 ± 0.2 vs. 2.7 ± 0.6, *p* = 0.036). Suggesting that the improvement in REM sleep caused by tDCS can be attributed to the increase in both the number of bouts and the prolonged duration of each bout. In ZT10–12, notable differences in wake bouts duration were observed (F_(2, 20)_ = 4.872, *p* = 0.019), with the Bex group exhibiting a significant increase compared to the baseline (3.9 ± 0.3 vs. 2.6 ± 0.3, *p* = 0.017). A trend towards increased NREM and REM bout numbers and duration was seen in the Bex + tDCS group.

During the assessment of NREM sleep percentage in the ZT14–16 period, a significant increase was observed in mice from the Bex + tDCS group compared to the Bex group. However, in the analysis of sleep architecture, despite a slight rise in both NREM bout number and duration in the tDCS group compared to the corresponding values in the Bex group (8.5 ± 1.8 vs. 4.7 ± 1.2 and 2.5 ± 0.2 vs. 2.4 ± 0.4), these differences did not reach statistical significance.

The observed effects suggest that tDCS may enhance both NREM and REM sleep, influencing bout number and duration, particularly evident in the acute stress response phase and ZT4. These findings contribute to a comprehensive understanding of tDCS effects on sleep architecture and its potential therapeutic role in insomnia.

#### 3.2.3. Alteration in NREM Delta Power

**Figure 9:**
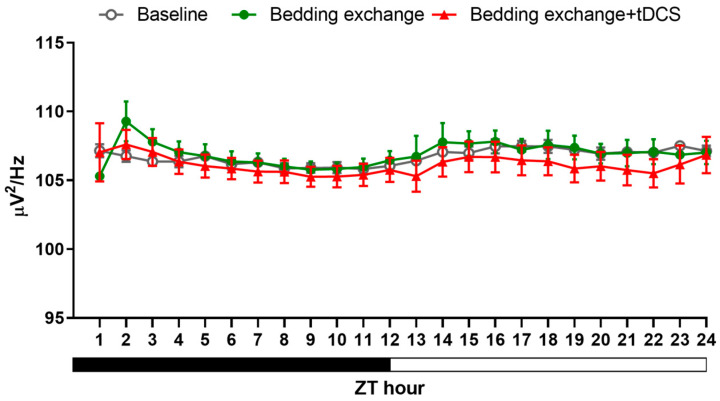
NREM delta power between different groups in the second experiment.

In conventional treatments for insomnia, pharmacotherapy can effectively extend overall sleep duration. However, these medications, as previously mentioned, may compromise sleep depth. Contrarily, our analysis of NREM delta power results (Figure 9) revealed no statistically significant differences among the three groups. This finding, on the one hand, supports the idea that tDCS does not diminish sleep depth. On the other hand, it suggests that the increased sleep effect induced by tDCS primarily stems from an augmentation of sleep quantity. In summary, the outcomes of this experiment not only affirm the efficacy of tDCS in reversing stress-induced acute insomnia but also indicate that this technique is unlikely to impact sleep quality.

### 3.3. Confirming the IL to VLPO Pathway in Mice

**Figure 10:**
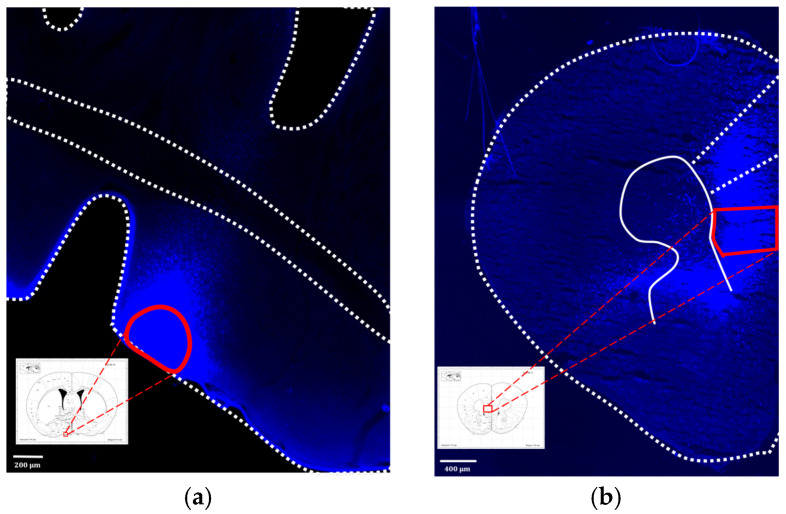
(**a**) Injection site of fluorogold, VLPO; (**b**) the neural tracer retrogradely traveled to the IL and its surrounding brain regions. The white lines represent the boundaries of different brain areas, and the area enclosed by the red line represents the VLPO in (**a**) and IL in (**b**).

To investigate whether the IL to VLPO neural pathway serves as the mechanism behind tDCS-induced sleep, we initially validated the existence of this pathway using fluorogold. Post-injection surgery, we allowed an eight-week period for retrograde labeling. The conclusive results, depicted in Figure 10, revealed that the brain region projecting to VLPO not only encompassed the anticipated IL area but also extended to other regions, such as the dorsal peduncular area and prelimbic area. This finding aligns with prior studies examining pathways in the rat brain utilizing cholera toxin subunit B (CTB) [53]. Our results affirm the presence of this pathway in the mouse brain, thereby enhancing confidence in manipulating this pathway using chemical modulation in subsequent experiments.

### 3.4. The Manipulation of the IL to VLPO Pathway

#### 3.4.1. Effect of tDCS after Specifically Blocking the IL-VLPO Pathway

After confirming the existence of the IL-VLPO pathway, we hypothesized that the effect of tDCS on amelioration of stress-induced insomnia is mediated by the activation of the IL-VLPO projection. To prove this hypothesis, the DREADD chemogenetic approach will be employed to selectively block the activity of IL-VLPO projection during the tDCS. In this experiment, mice underwent virus injection, followed by EEG and tDCS electrode implantation through stereotaxic surgery. To mitigate potential biases, the order of interventions was randomized, and a waiting period of at least one week between successive procedures was implemented. This approach ensured that the animals did not experience a reduction in the efficacy of insomnia intervention due to habituation to bedding exchange.

**Figure 11:**
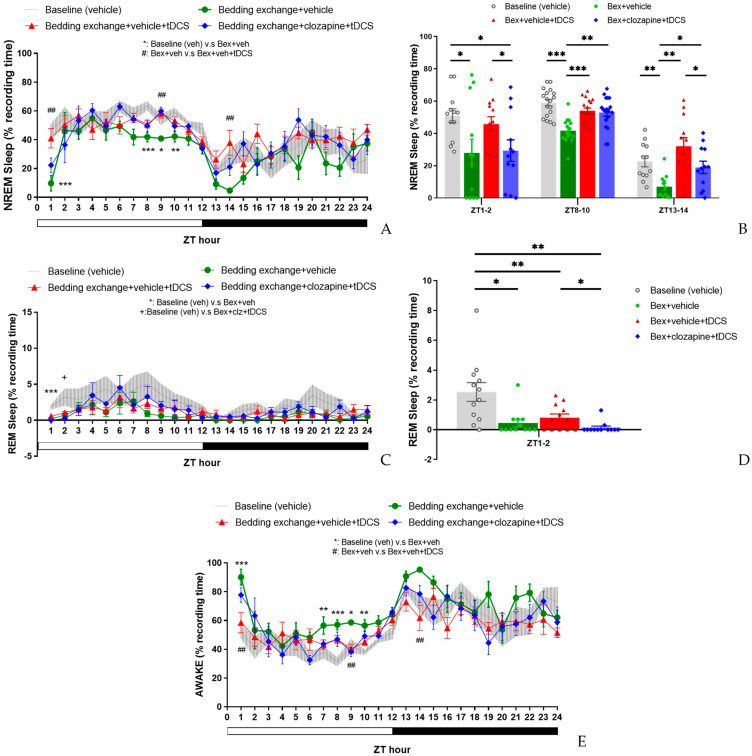
The impact of tDCS after inhibiting the IL to VLPO circuit. (**A**) The decrease in NREM sleep in mice caused by bedding exchange primarily occurs in ZT1, 8, 9, and 10, with no statistical difference between Bex + veh + tDCS and Bex + clz + tDCS group; (**B**) During the ZT1−2, ZT8−10, and ZT13−14 periods, the Bex group consistently exhibited significantly lower NREM sleep compared to the baseline. The Bex + veh + tDCS group demonstrated a reversal of insomnia effects at ZT8−10 and ZT13−14, while Bex + clz + tDCS reduced the effect of tDCS at ZT1−2 and ZT13−14; (**C**) Mice in all the group that under bedding exchange exhibited a significant lower REM sleep at ZT1, and in ZT2, Bex + clz + tDCS was the only group that statistical lower than baseline; (**D**) When analyzed over the ZT1–2 period, the Bex + clz + tDCS group exhibited a lower percentage of REM sleep compared to the Bex + veh + tDCS group. This observation indicates that tDCS loses its efficiency in reversing stress-induced insomnia after inhibiting the IL-VLPO pathway; (**E**) Mice in Bex + veh group exhibited higher wake percentage at ZT1, 7, 8, 9, and 10. And mice in the Bex + veh + tDCS group displayed the therapeutic effect at ZT1, 9, and 14. (* *p* < 0.05, ** *p* < 0.01, *** *p* < 0.001: Baseline (veh) vs. Bex+veh, ## *p* < 0.01: Bex + veh vs. Bex + veh + tDCS, + *p* < 0.05: Baseline (veh) vs. Bex + clz + tdcs ; Repeated-measures ANOVA followed by the Bonferroni test was conducted in (**A**,**C**,**E**); * *p* < 0.05, ** *p* < 0.01, *** *p* < 0.001; Paired *t*-test was conducted in (**B**,**D**)). Data are presented as the mean ± SEM. *n* = 6; ZT: zeitgeber time.

In assessing the percentage of NREM sleep (Figure 11A), we examined the effects among four experimental groups [baseline (veh), Bex + veh, Bex + veh + tDCS, Bex + clz + tDCS] during ZT1–12 and ZT13–24 time periods. The results of the analysis indicated no significant interaction between time and group in both cycles. Exploring group differences, we observed variations among the groups during the light cycle (F_(3, 20)_ = 14.549, *p* < 0.001), while no differences were observed during the dark cycle. Post hoc analyses of group main effects at various time points revealed a significant decrease in NREM sleep proportion for the Bex + veh group compared to the baseline group at ZT1 (*p* = 0.001) and ZT8, ZT9, and ZT10 (*p* = 0.001, *p* = 0.034, *p* = 0.009, respectively). This result aligns with the two phases of acute stress response and acute insomnia observed in previous experiments, although the timing is not entirely consistent, likely attributed to individual differences among the mouse groups. The Bex + veh + tDCS group exhibited a significant improvement in NREM sleep at ZT1 (*p* = 0.004), ZT9 (*p* = 0.004), and ZT14 (*p* = 0.006) compared to the Bex + veh group, reaching a level that showed no statistical difference from the baseline group in these three time periods. Regarding the Bex + clz + tDCS group, no statistical difference was observed at ZT1 compared to the Bex + veh group or the Bex + veh + tDCS group. However, at ZT9, a significant improvement in NREM sleep proportion was observed compared to the Bex + veh group (*p* = 0.002).

While we anticipated that inhibiting the IL-VLPO pathway would reduce the effectiveness of tDCS, no significant suppression of tDCS was observed. We attribute this result to the conservative nature of the Bonferroni correction in masking the inhibitory effects of the pathway. Consequently, we conducted paired *t*-tests among all groups to investigate the potential impact of clozapine-mediated inhibition of the IL-to-VLPO pathway on the efficacy of tDCS (Figure 11B). The results indicated a significant reduction in the enhancement of NREM sleep following pathway inhibition at ZT1–2 (t_(11)_ = 2.247, *p* = 0.046) and ZT13–14 (t_(11)_ = 2.283, *p* = 0.043), with no significant differences observed at ZT8–10.

Upon scrutinizing the results of REM sleep (Figure 11C), statistical analysis for both the light and dark cycles revealed no significant interaction between group and time factors, and no differences between groups were observed in either cycle. However, a detailed examination of the main effects of groups at each time point uncovered a noteworthy decrease in REM sleep percentage at ZT1 for all groups subjected to bedding exchange compared to the baseline group (*p* < 0.001). At ZT2, the group receiving Bex + clz + tDCS continued to exhibit a significantly lower REM percentage compared to the baseline group, while no statistical differences were observed among the other groups.

In light of these observed differences, we conducted paired *t*-tests for the groups within the ZT1–2 time period. The results showed no significant difference between the Bex + veh + tDCS group and the Bex group, consistent with observations in the second experiment. However, when comparing the Bex + clz + tDCS group with the Bex + veh + tDCS group, a significant decrease in REM sleep percentage was observed (t_(11)_ = 2.411, *p* = 0.035) (Figure 11D). This result suggests that inhibiting the IL-to-VLPO pathway may have a certain impact on the effectiveness of tDCS on REM sleep.

In the statistical analysis of the percentage of wakefulness (Figure 11E), no significant interaction was observed between group and time factors in both light and dark cycles. Noteworthy group differences emerged only in the light cycle (F_(3, 20)_ = 15.144, *p* < 0.001), with no significant variations in the dark cycle. Post hoc comparisons of the group’s main effect at each time point revealed that the Bex + veh group exhibited significantly higher wakefulness percentages compared to the baseline group at ZT1 (*p* = 0.001), ZT7 (*p* = 0.037), ZT8 (*p* = 0.001), ZT9 (*p* = 0.023), and ZT10 (*p* = 0.003). When comparing the Bex + veh + tDCS group with the Bex + veh group, a significant reduction in wakefulness percentage was observed at ZT1 (*p* = 0.004), ZT9 (*p* = 0.009), and ZT14 (*p* = 0.006), consistent with the time points observed in the NREM sleep results. However, no statistical differences were observed between the Bex + veh + tDCS group and the Bex + clz + tDCS group at various time points, possibly aligning with our discussion in the NREM sleep results. This suggests that the inhibitory effects of clozapine on the IL-to-VLPO pathway may not significantly influence wakefulness when combined with tDCS, as reflected in the comparable outcomes between the Bex + veh + tDCS and Bex + clz + tDCS groups at different time points.

#### 3.4.2. Alterations in Sleep Architecture and Quality

In the analysis of sleep architecture (Table 3), distinct patterns emerged during the light cycle, particularly in the wake bout duration. The Bex + veh group exhibited a significantly higher wake bout duration than the other three groups, while no statistical differences were observed among the remaining groups. Within the dark cycle, the only identified statistical difference was between the Bex + veh + tDCS and Bex + veh groups. Notably, in the comparison of NREM, the former showed a higher bout number (10.4 ± 0.6 vs. 5.7 ± 1.1, *p* = 0.017). Conversely, in the wake comparison, the Bex + veh + tDCS group demonstrated a significantly lower bout duration (5.6 ± 0.6 vs. 16.5 ± 2.9, *p* = 0.007) and a higher bout number (7.6 ± 0.6 vs. 4.4 ± 0.7, *p* = 0.01).

Further dissecting structural differences within each time period, during ZT1–2, the NREM bout number in the Bex + veh + tDCS group significantly increased compared to that of Bex + veh (12.0 ± 1.7 vs. 3.5 ± 1.0, *p* = 0.001), while the Bex + clz + tDCS group (5.8 ± 1.0) reversed this change (*p* = 0.012). In REM sleep structure, Bex + veh exhibited significantly lower bout number compared to baseline (0.3 ± 0.2 vs. 1.9 ± 0.5, *p* = 0.004), and Bex + clz + tDCS showed lower bout number and bout duration compared to baseline (*p* = 0.003, *p* = 0.007), but these changes did not reach statistical significance when compared to Bex + veh + tDCS. In the wake structure, Bex + veh + tDCS showed significantly lower bout duration (4.2 ± 0.8 vs. 30.5 ± 8.1, *p* = 0.007) and higher bout number (9.2 ± 1.1 vs. 2.7 ± 0.7, *p* < 0.001) compared to Bex + veh. When compared to Bex + veh + tDCS, Bex + clz + tDCS exhibited a significant decrease in bout number (5.4 ± 0.9 vs. 9.2 ± 1.1, *p* = 0.027), while bout duration increased without statistical significance (13.1 ± 5.7 vs. 4.2 ± 0.8).

During the ZT8–10 time period, no statistical differences were observed among the three groups outside the baseline in both NREM and REM sleep architectures. In the architecture of wake, Bex + veh + tDCS exhibited significantly lower bout duration compared to Bex + veh (2.0 ± 0.2 vs. 3.1 ± 0.2, *p* = 0.005). When compared to Bex + clz + tDCS, Bex + veh + tDCS showed a higher bout number (13.2 ± 1.3 vs. 9.6 ± 0.3, *p* = 0.016).

In the ZT13–14 time period, Bex + veh + tDCS significantly increased NREM bout number compared to that of Bex + veh (8.9 ± 1.6 vs. 1.8 ± 0.4, *p* = 0.004). While Bex + clz + tDCS showed a slight decrease compared with tDCS enhancing NREM bout number (6.0 ± 1.7), the difference did not reach statistical significance when compared to Bex + veh + tDCS. In the wake architecture, Bex + veh + tDCS significantly increased bout number (6.3 ± 0.8 vs. 1.8 ± 0.3, *p* = 0.006) and significantly decreased bout duration (7.7 ± 2.6 vs. 28.6 ± 5.1, *p* = 0.01) compared to Bex + veh. No statistical differences were observed between the Bex + clz + tDCS group and the other groups.

**Figure 12:**
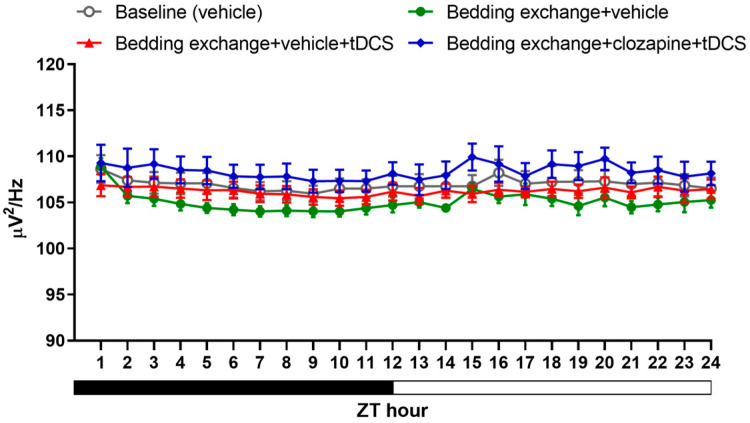
NREM delta power obtained from experiment 3.

As for the result of NREM delta power (Figure 12), although there are slight differences in the average delta power in each manipulation group, no statistical differences were observed between the groups at any time point. This result indicates that the increased sleep induced by tDCS is not manifested through the enhancement of NREM power. Additionally, it suggests that inhibiting the neural pathway does not significantly impact sleep quality.

#### 3.4.3. Fluorescence Image of Brain Slices after Microinjection of Virus

**Figure 13:**
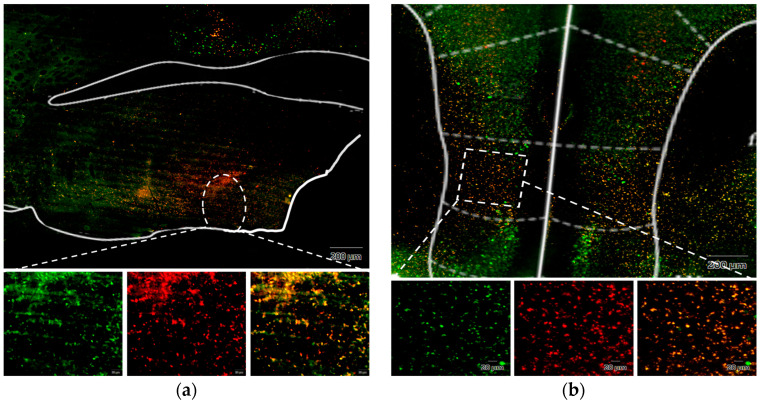
Fluorescence image of brain slices after microinjection of virus. (**a**) Injection site in VLPO; (**b**) injection site in IL. The enlarged images in the lower panel, from left to right, depict the green fluorescence presented by GFP, the red fluorescence exhibited by mCherry, and the merged image displaying a vibrant orange color resulting from the overlay of both. The white lines represent the boundaries of other structural regions in the brain.

To verify the successful viral infection at the targeted site, mice were euthanized following the completion of all experimental recordings, and their heads were harvested for brain slice preparation. Under fluorescence microscopy, the virus administered into the ventrolateral preoptic nucleus was marked by the mCherry red fluorescent protein (Figure 13A), whereas the virus introduced into the IL region expressed the green fluorescent protein (GFP) (Figure 13B), both distinctly localized. Upon merging these images, cells co-infected with both viruses exhibited a vibrant orange fluorescence. This outcome conclusively establishes the effective suppression of the IL to VLPO pathway during clozapine administration. However, the observed inhibitory effect does not entirely align with our expectations, a point we will further explore in the subsequent discussion.

## 4. Discussion

This study investigates the impact of tDCS on stress-induced insomnia through three experimental phases. Additionally, it delves into the role of the neural pathway from the IL to the VLPO. The selected current intensity (0.06 mA) for tDCS was deliberately lower than commonly employed in animal studies [34]. This decision stemmed from two primary considerations. Firstly, the impact of tDCS does not demonstrate a linear correlation with an escalation in current intensity. Secondly, elevated currents have the potential to cause discomfort in animals, manifesting as itching or disruption of ongoing activities. Notably, the choice of current intensity was also influenced by the placement of the reference electrode; in this study, we opted for EEG placement above the cerebellum as opposed to methods like chest patching.

The outcomes of our experiment revealed a noteworthy augmentation in the percentage of NREM sleep for both naïve and stress-induced insomnia mice. This consistent trend across both experiments provides preliminary confirmation of the potential efficacy of tDCS in promoting sleep. Notably, tDCS exhibited an influence on REM sleep, particularly in the post-stimulation hours, where there was a significant upsurge in the percentage of REM sleep. Crucially, the durability of these effects for over 12 h post-stimulation suggests that the mechanism of tDCS in promoting sleep may encompass not only immediate impacts but also the modulation of neural plasticity, leading to more enduring effects [62,63].

In the stress-induced insomnia mice, changes in NREM sleep were delineated into two stages: the acute stress response stage and the acute insomnia stage. In both stages, the ameliorative effect of tDCS on NREM sleep was corroborated, primarily characterized by an increase in sleep duration rather than alterations in depth. Conversely, REM sleep exhibited a gradual rise in its proportion within the initial 3–4 h post-electrical stimulation, followed by a lack of significant effects. In summary, these findings underscore the potential effectiveness of tDCS as a non-pharmacological intervention for sleep, presenting promising prospects for clinical applications.

We substantiated the existence of the IL to VLPO projection pathway in the mouse brain using Fluorogold labeling. This validation revealed not only the anticipated upper edge region but also additional areas, laying a robust foundation for future investigations into modulating tDCS effects through chemical interventions within this pathway. While IL is typically associated with behavioral regulation, such as fear conditioning [64], reward processing [65], and habit learning [66], we selected this pathway due to the widespread use of the DLPFC in human tDCS studies, despite the lack of a direct equivalent in mice [48,49]. Some studies suggest that the prelimbic and infralimbic areas in rodents may serve as a model for the human DLPFC [47].

Experimental results inhibiting the IL to VLPO projection pathway indicated that tDCS could enhance NREM sleep even when the pathway was suppressed during the ZT8–10 period. However, a certain degree of attenuation was observed at the initial time points (ZT1–2). The limited inhibitory effects may be associated with the duration of clozapine, as studies propose that clozapine reaches peak blood concentration 15 min after intraperitoneal injection, rapidly decreasing thereafter [55]. Consequently, tDCS initiation 15 min after clozapine administration aimed to synchronize the interventions. Nonetheless, the rapid metabolism of clozapine might hinder sustained inhibition of the after-effects post-tDCS. This could elucidate the significantly lower NREM sleep in the inhibited pathway group compared to the non-inhibited group during the initial acute stress response period (ZT1–2), with no statistical difference during the subsequent acute insomnia period (ZT8–10). In summary, these experimental results suggest that the IL to VLPO projection pathway plays a modulating role in the sleep improvement induced by tDCS, particularly concerning NREM sleep.

A distinctive aspect of the third experiment was the absence of changes in REM sleep in the initial hours, irrespective of pathway inhibition. Across all experimental groups, no statistical differences in REM proportions were observed after ZT3. This variability could be attributed to individual differences among mouse groups, akin to the inconsistency in the sleep decline period exhibited by the Bex group in experiments two and three.

## 5. Conclusions

In conclusion, our study establishes parameter settings that exert a positive impact on sleep in mice. The tDCS, with anodal stimulation in the frontal lobe, significantly alters the quantity and duration of NREM sleep. However, the effects on REM sleep may necessitate further exploration with increased sample sizes and additional experiments. In discussing potential underlying mechanisms, we elucidated that the IL to VLPO pathway indeed plays a regulatory role, but further research is needed to fully comprehend its involvement in tDCS. Overall, through these comprehensive studies, we deepen our understanding of the effects of tDCS on sleep, providing valuable insights for future research and clinical applications in stress-induced insomnia therapy.

## 6. Limitation

In this study, there are certain limitations. We could only confirm the intensity of incoming and outgoing electrical currents through external electrodes. However, the actual flow of current within the brain, once it enters, is beyond our observation and control. Achieving precise activation of specific brain regions with tDCS remains a challenge. Consequently, the observed pathway from IL to VLPO in the effectiveness of tDCS on sleep improvement might represent only a partial explanation. Due to the inability to directly observe how the current disperses within the brain, we cannot exclude the potential involvement of other pathways. Nevertheless, our results still provide evidence for the involvement of the infralimbic area as one pathway in tDCS and offer insights into its potential role in treating insomnia.

## Figures and Tables

**Table 1:** Sleep architecture obtained from experiment 1.

Group	ZT Hour	L:D Cycle	Bout Number			Bout Duration		
			NREM	WAKE	REM	NREM	WAKE	REM
Control	13–24	D	9.5 ± 2.0	6.7 ± 1.5	1.1 ± 0.2	2.1 ± 0.4	10.2 ± 3.7	0.5 ± 0.1
tDCS	13–24	D	8.4 ± 1.4	5.9 ± 0.7	1.1 ± 0.1	3.0 ± 0.2 *	8.0 ± 2.2	0.6 ± 0.1
Control	1′–12′	L	14.3 ± 2.0	9.7 ± 1.3	1.9 ± 0.5	2.4 ± 0.4	2.9 ± 0.5	1.0 ± 0.2
tDCS	1′–12′	L	12.3 ± 1.3	8.2 ± 0.9	2.1 ± 0.2	3.2 ± 0.4	2.7 ± 0.3	1.2 ± 0.2
Control	13–15	D	8.3 ± 1.7	5.9 ± 1.3	0.3 ± 0.1	1.6 ± 0.1	12.0 ± 3.8	0.3 ± 0.1
tDCS	13–15	D	9.6 ± 1.6	6.1 ± 0.8	1.3 ± 0.2 *	3.8 ± 0.7 *	4.5 ± 0.6	0.7 ± 0.1
Control	4′–12′	L	15.3 ± 2.0	10.4 ± 1.4	1.8 ± 0.6	2.1 ± 0.3	2.6 ± 0.4	1.0 ± 0.2
tDCS	4′–12′	L	13.2 ± 1.2	8.5 ± 0.9	2.2 ± 0.1	3.0 ± 0.3 *	2.3 ± 0.2	1.3 ± 0.2

* *p* < 0.05, Data are presented as the mean ± SEM. *n* = 5.

**Table 2:** Sleep architecture obtained from experiment 2.

Group	ZT Hour	L:D Cycle	Bout Number			Bout Duration		
			NREM	WAKE	REM	NREM	WAKE	REM
Control	1–12	L	13.8 ± 1.4	9.5 ± 0.9	1.9 ± 0.2	2.6 ± 0.2	3.1 ± 0.4	1.0 ± 0.1
Bex	1–12	L	11.2 ± 0.9	7.9 ± 0.5	1.2 ± 0.3	2.2 ± 0.2	8.7 ± 1.3 **	0.6 ± 0.2
Bex + tDCS	1–12	L	13.4 ± 1.3	8.5 ± 0.9	1.9 ± 0.4	2.3 ± 0.2	5.6 ± 1.4	1.0 ± 0.2
Control	13–24	D	8.6 ± 1.2	6.3 ± 0.9	1.0 ± 0.1	2.2 ± 0.2	12.2 ± 3.1	0.6 ± 0.1
Bex	13–24	D	7.8 ± 1.0	5.5 ± 0.8	0.6 ± 0.1 *	2.2 ± 0.3	12.3 ± 2.1	0.4 ± 0.1
Bex + tDCS	13–24	D	9.4 ± 1.3	6.6 ± 0.9	0.9 ± 0.1	2.3 ± 0.2	10.1 ± 3.1	0.6 ± 0.1
Control	1–2	L	12.9 ± 1.9	7.9 ± 0.9	2.0 ± 0.2	2.9 ± 0.3	3.3 ± 0.5	1.0 ± 0.1
Bex	1–2	L	2.2 ± 0.7 *	1.8 ± 0.5 **	0.3 ± 0.2 ***	1.9 ± 0.6	34.5 ± 7.0 ***	0.1 ± 0.1 **
Bex + tDCS	1–2	L	7.5 ± 1.8	5.6 ± 1.6	0.4 ± 0.3 ***	2.0 ± 0.2	19.2 ± 7.0	0.3 ± 0.2 **
Control	4	L	14.7 ± 1.4	11.2 ± 1.0	2.2 ± 0.4	2.3 ± 0.3	2.2 ± 0.3	0.9 ± 0.1
Bex	4	L	11.3 ± 1.5	7.8 ± 0.9	1.8 ± 0.7	3.2 ± 0.4	2.7 ± 0.6	0.6 ± 0.2
Bex + tDCS	4	L	14.8 ± 1.9	7.7 ± 0.7	4.5 ± 0.9 ^+^	2.9 ± 0.4	1.1 ± 0.2 ^+^	1.4 ± 0.2 ^+^
Control	10–12	L	15.0 ± 1.3	10.8 ± 0.8	1.3 ± 0.3	2.1 ± 0.2	2.6 ± 0.3	0.7 ± 0.2
Bex	10–12	L	13.2 ± 0.9	9.7 ± 0.6	1.1 ± 0.4	1.6 ± 0.1	3.9 ± 0.3 *	0.6 ± 0.2
Bex + tDCS	10–12	L	15.9 ± 1.7	10.5 ± 1.0	1.6 ± 0.5	1.8 ± 0.1	2.9 ± 0.4	1.0 ± 0.3
Control	14–16	D	7.6 ± 1.4	5.3 ± 0.9	0.8 ± 0.2	1.8 ± 0.2	15.3 ± 4.7	0.5 ± 0.1
Bex	14–16	D	4.7 ± 1.2	3.2 ± 0.8	0.4 ± 0.1	2.4 ± 0.4	18.2 ± 2.9	0.2 ± 0.1
Bex + tDCS	14–16	D	8.5 ± 1.8	5.6 ± 1.0	0.6 ± 0.3	2.5 ± 0.2	9.3 ± 2.5	0.5 ± 0.3

* *p* < 0.05, ** *p* < 0.01, *** *p* < 0.001 vs. Control (baseline), ^+^ *p* < 0.05 vs. Bex. Data are presented as the mean ± SEM. *n* for the baseline, Bex, and Bex + tDCS groups are 11, 6, and 6, respectively.

**Table 3:** Sleep architecture obtained from experiment 3.

Group	ZT Hour	L:D Cycle	Bout Number			Bout Duration		
			NREM	WAKE	REM	NREM	WAKE	REM
Control	1–12	L	11.8 ± 0.6	7.6 ± 0.3	1.9 ± 0.6	2.9 ± 0.2	3.4 ± 0.3 **	0.7 ± 0.1
Bex + veh	1–12	L	11.5 ± 0.4	8.5 ± 0.3	0.9 ± 0.4	2.3 ± 0.2	8.3 ± 1.2	0.4 ± 0.2
Bex + veh + tDCS	1–12	L	14.3 ± 1.3	10.3 ± 1.1	1.3 ± 0.3	2.2 ± 0.2	3.2 ± 0.4 **	0.4 ± 0.1
Bex + clz + tDCS	1–12	L	12.0 ± 0.8	8.4 ± 0.7	1.1 ± 0.2	2.6 ± 0.3	4.8 ± 1.0 *	0.6 ± 0.1
Control	13–24	D	6.8 ± 0.6	5.2 ± 0.4	1.0 ± 0.2	2.6 ± 0.2	11.2 ± 1.7	0.4 ± 0.1
Bex + veh	13–24	D	5.7 ± 1.1	4.4 ± 0.7	0.3 ± 0.1	2.4 ± 0.3	16.5 ± 2.9	0.1 ± 0.1
Bex + veh + tDCS	13–24	D	10.4 ± 0.6 *	7.6 ± 0.6 *	0.7 ± 0.2	2.1 ± 0.2	5.6 ± 0.6 *	0.3 ± 0.1
Bex + clz + tDCS	13–24	D	7.2 ± 1.4	5.5 ± 0.7	0.7 ± 0.2	2.7 ± 0.2	10.9 ± 2.2	0.3 ± 0.1
Control	1–2	L	9.3 ± 1.1 *	5.9 ± 0.7	1.9 ± 0.5 **	3.4 ± 0.3	4.6 ± 0.7 **	0.7 ± 0.1
Bex + veh	1–2	L	3.5 ± 1.0	2.7 ± 0.7	0.3 ± 0.2	3.0 ± 0.9	30.5 ± 8.1	0.2 ± 0.1
Bex + veh + tDCS	1–2	L	12.0 ± 1.7 **	9.2 ± 1.1 ***	0.8 ± 0.3	2.2 ± 0.2	4.2 ± 0.8 **	0.4 ± 0.1
Bex + clz + tDCS	1–2	L	5.8 ± 1.0 ^#^	5.4 ± 0.9 ^#^	0.2 ± 0.2 ^++^	2.9 ± 0.8	13.1 ± 5.7	0.0 ± 0.0 ^++^
Control	8–10	L	13.6 ± 1.1	8.5 ± 0.5 ^##^	2.3 ± 0.7	2.8 ± 0.3 **	2.4 ± 0.2	0.7 ± 0.2
Bex + veh	8–10	L	15.2 ± 1.0	11.5 ± 0.3	0.7 ± 0.2	1.6 ± 0.2	3.1 ± 0.2	0.4 ± 0.1
Bex + veh + tDCS	8–10	L	17.7 ± 1.4	13.2 ± 1.3	1.4 ± 0.2	1.9 ± 0.2 ^+^	2.0 ± 0.2 **	0.6 ± 0.2
Bex + clz + tDCS	8–10	L	14.9 ± 0.7	9.6 ± 0.3 ^##^	1.2 ± 0.4	2.2 ± 0.1	2.6 ± 0.2	0.8 ± 0.3
Control	13–14	D	6.6 ± 0.9	4.4 ± 0.9	0.3 ± 0.2	2.0 ± 0.3	12.4 ± 2.3	0.2 ± 0.1
Bex + veh	13–14	D	1.8 ± 0.4	1.8 ± 0.3	0.0 ± 0.0	1.8 ± 0.5	28.6 ± 5.1	0.0 ± 0.0
Bex + veh + tDCS	13–14	D	8.9 ± 1.6 **	6.3 ± 0.8 **	0.5 ± 0.3	1.9 ± 0.3	7.7 ± 2.6 *	0.2 ± 0.1
Bex + clz + tDCS	13–14	D	6.0 ± 1.7	4.4 ± 1.0	0.4 ± 0.2	1.7 ± 0.4	16.2 ± 5.3	0.3 ± 0.1

* *p* < 0.05, ** *p* < 0.01, *** *p* < 0.001 vs. Bex + veh, ^#^ *p* < 0.05, ^##^ *p* < 0.01 vs. Bex + veh + tDCS, ^+^ *p* < 0.05, ^++^ *p* < 0.01 vs. control (baseline). Data are presented as the mean ± SEM. *n* = 6.

## Data Availability

The data presented in this study are available on request from the corresponding author. The data are not publicly available due to the huge and complicated dataset.
